# TRAJECTORIES OF SKELETAL MUSCLE MASS AND FAT MASS AND THEIR IMPACTS ON MORTALITY IN OLDER JAPANESE ADULTS

**DOI:** 10.1093/geroni/igac059.2125

**Published:** 2022-12-20

**Authors:** Satoshi Seino, Yu Taniguchi, Miki Narita, Takumi Abe, Yu Nofuji, Yuri Yokoyama, Shoji Shinkai, Yoshinori Fujiwara

**Affiliations:** Tokyo Metropolitan Institute of Gerontology, Itabashi, Tokyo, Japan; National Institute for Environmental Studies, Tsukuba, Ibaraki, Japan; Tokyo Metropolitan Institute of Gerontology, Itabashi, Tokyo, Japan; Tokyo Metropolitan Institute of Gerontology, Itabashi, Tokyo, Japan; Tokyo Metropolitan Institute of Gerontology, Itabashi, Tokyo, Japan; Tokyo Metropolitan Institute of Gerontology, Itabashi, Tokyo, Japan; Kagawa Nutrition University, Sakado, Saitama, Japan; Tokyo Metropolitan Institute of Gerontology, Tokyo, Tokyo, Japan

## Abstract

Skeletal muscle mass and fat mass have differential impacts on mortality between men and women. We aimed to determine age-related trajectories of skeletal muscle mass index (SMI) and fat mass index (FMI) among men and women and to examined their impacts on mortality risks. This prospective study included 1,770 (863 men and 907 women) aged ≥65 years who participated in health check-ups; the total number of observations was 6,110. SMI and FMI were determined using segmental multi-frequency bioelectrical impedance analysis, and their age-related trajectories from age 65–90 years were examined using group-based semiparametric mixture models. SMI and FMI age-related trajectories for all-cause mortality were determined by multivariate-adjusted hazard ratios (HRs) and 95% confidence intervals (CIs). SMI and FMI trajectories were classified into three trajectories in both sexes: the low- (28.6% and 34.0%), middle- (56.0% and 47.5%), and high-trajectory (15.4% and 18.5%) groups in men and the low- (27.6% and 40.9%), middle- (51.6% and 48.1%), and high-trajectory (20.8% and 11.0%) groups in women. The median follow-up was 5.3 years; 101 (11.7%) men and 56 (6.2%) women died. Compared with the low-trajectory groups, male multivariate-adjusted HRs for mortality in the middle- and high-trajectory groups were 0.89 (95% CI: 0.57–1.39) and 0.34 (0.13–0.93) for SMI, and 0.76 (0.47–1.23) and 1.13 (0.60–2.14) for FMI, respectively. Corresponding female multivariate-adjusted HRs were 1.00 (0.50–2.02) and 1.64 (0.62–4.36) for SMI, and 0.74 (0.38–1.43) and 0.37 (0.12–1.14) for FMI. Maintaining high skeletal muscle mass is important for prolonging life expectancy, especially in men.

